# Lipid Oxidation Products on Inflammation-Mediated Hypertension and Atherosclerosis: A Mini Review

**DOI:** 10.3389/fnut.2021.717740

**Published:** 2021-09-30

**Authors:** Xin-Fang Leong

**Affiliations:** Department of Craniofacial Diagnostics and Biosciences, Faculty of Dentistry, Universiti Kebangsaan Malaysia, Kuala Lumpur, Malaysia

**Keywords:** atherosclerosis, cooking oil, heating, hypertension, inflammation, lipid peroxidation

## Abstract

Cardiovascular diseases such as hypertension and atherosclerosis are the common causes of mortality in developed and developing countries. Repeated heating of the dietary oil is a common practice to reduce cost during food preparation. When the cooking oil is heated at high temperatures, production of free radicals augments the oxidative degradation of lipids and depletes the natural antioxidant contents of the cooking oil. Chronic intake of foods prepared using reheated oil could impair antioxidant capacity, leading to oxidative stress and inflammation. This review aims to summarize the current evidence of lipid oxidation products on hypertension and atherosclerosis via inflammatory pathway. In particular, toxic lipid oxidation products such as malondialdehyde and 4-hydroxy-2-nonenal are taken into account. Understanding the signaling pathways underlying the pathology associated with the lipid oxidation-derived aldehydes may be useful to develop therapeutic strategies for the prevention of inflammatory-related cardiovascular complications.

## Introduction

Deep frying is a common cooking method for food preparation. The flavor and crispness of fried food provide a sensory preference for many consumers. Frying involves heat and mass transfer (1). In addition, there will be a change in physical and chemical reactions during the frying process. Factors such as temperature, duration of heating, ratio of unsaturated to saturated fatty acids in cooking oil, type of cooking oil, presence of prooxidants and/or antioxidants are important to determine the quality of cooking oil. The habit of using reheated cooking oil during food preparation is not only restricted to food stalls but include restaurants with the aim to reduce costs. When the oil is heated to high temperatures and is used repeatedly, physical reactions such as formation of foam, darkening of oil color, increased viscosity and production of off-flavor in oil occur. These reactions may impair the organoleptic qualities, including the taste and smell, and the nutritional value of the fried food.

When the cooking oil undergoes repeated heating, chemical reactions such as hydrolysis, oxidation and polymerization may affect the chemical structure of triacylglycerol molecules with the polyunsaturated fatty acid (PUFA) molecules mostly altered (2). Hydroperoxides are primary product of lipid oxidation. They are unstable intermediates and rapidly broken down into secondary products such as hydrocarbons, alcohols, aldehydes and ketones due to oxidation and hydrolysis reactions. Polymerization process of hydrolysis products is enhanced in the presence of high temperatures, forming cyclic fatty acid monomers, dimers and polymers. The generated lipid peroxidation products have been known to cause changes in the physiochemical properties of fats. It was demonstrated that antioxidants such as vitamin E were subjected to degradation as the frying episodes increased (3), which may contribute to a reduction in oxidative stability. Hydroperoxides and aldehydes are formed when the cooking oil is heated. These toxic products are then absorbed by the fried food, and gain access to the gastrointestinal system and later to systemic circulation when the food is to be consumed (4).

Free radicals generated during frying process may damage lipid membranes by initiating lipid peroxidation. Arachidonic acid is found abundantly in the biological membranes. With the high number of double bonds present, arachidonic acid becomes the target of lipid peroxidation. Malondialdehyde (MDA) and 4-hydroxy-2-nonenal (4-HNE) are generated following degradation of lipid peroxides. Hydroperoxides can be broken down into ketones, and eventually forming MDA (5). 4-HNE is an example of hydroxy alkenals. It is formed following peroxidation of n-6 PUFAs (6). These α, β-unsaturated aldehydes are reactive with proteins, phospholipids, and nucleic acids. It has been documented that these major secondary oxidation end products of peroxidized PUFA play vital role in the development of oxidative stress-related inflammatory pathologies such as hypertension, atherosclerosis, diabetes, metabolic syndrome, and cancer (7–9).

## Effect of Thermally Oxidized Cooking Oil on Inflammation

Several studies were conducted to evaluate the effect of heated cooking oil on inflammation using animal models (Table 1). Studies have shown that rats fed with repeatedly heated palm oil (10) or soy oil (11) increased expression of adhesion molecules in aorta of rats in comparison to fresh or heated once vegetable oils. Furthermore, the authors reported that there was a positive correlation between expression of adhesions molecules and blood pressure (BP).

**Table 1:** Effect of heated culinary oil on inflammation.

**Author (Reference)**	**Type of oil**	**Feeding duration**	**Study model**	**Findings**
Ng et al. (10)	Repeatedly heated palm oil	24 weeks	Adult male Sprague-Dawley rats	↑ VCAM-1
Ng et al. (11)	Repeatedly heated soybean oil	24 weeks	Adult male Sprague-Dawley rats	↑ ICAM-1, VCAM-1
Hamsi et al. (12)	Repeatedly heated virgin coconut oil	24 weeks	Adult male Sprague-Dawley rats	↑ ICAM-1, VCAM-1, C-RP
Bogariani & Sudiarta (13)	Used cooking oil	10 weeks	Adult male Wistar rats	↑ IL-6, TNF-α
Mboma et al. (14)	CFAM and soybean oil	28 days	Young male Wistar rats	↑ IL-6
Tan et al. (15)	Moderately and highly heated soybean oil	21 days	Young female broiler chickens	↑ IL-22
Zhang et al. (16)	Repeatedly heated canola oil or polar compounds	4 weeks	Young C57BL/6 male mice	↑ TNF-α, IL-1β, IL-6, MCP-1, IFN-γ

*Cyclic fatty acid monomers (CFAM), C-reactive protein (C-RP), Intracellular adhesion molecule-1 (ICAM-1), Interferon-gamma (IFN-γ), Interleukin (IL), Monocyte chemoattractant protein-1 (MCP-1), Tumor necrosis factor-alpha (TNF-α) and Vascular adhesion molecule-1 (VCAM-1)*.

Hamsi et al. (12) studied the effect of heated virgin coconut oil on rats for 6 months of feeding duration. It was demonstrated that repeatedly heated virgin coconut oil elevates inflammatory markers and thromboxane together with a reduction of prostacyclin level in plasma. Thromboxane and prostacyclin are both prostanoids with a regulatory role in BP. Based on a study by Bogoriani & Sudiarta (13), prolonged feeding of used cooking oil was found to cause inflammation in rats as indicated by increased plasma levels of inflammatory mediator and MDA.

Mboma et al. (14) conducted research using cyclic fatty acid monomers (CFAM) from heated linseed oil on oxidative stress and inflammation. They fed the rats with a combination of CFAM and canola oil or CFAM with soybean oil. The findings showed that CFAM together with soybean oil had increased inflammation and oxidative stress as compared to CFAM in combination with canola oil. It was suggested that soybean oil contains higher percentage of PUFAs whereas canola oil is rich in oleic acid, the most abundant type of monounsaturated fatty acids in diet. Soybean oil with a greater number of double bonds as found in linoleic acid, is prone to oxidative damage than canola oil.

Tan et al. (15) fed 1-day old broiler chicken with fresh soybean oil or soybean oil oxidized at various levels. It was found that moderately and highly oxidized soybean oil had increased ileal level of mRNA expression of interleukin (IL)-22. In addition, oxidized soybean oil caused increased intestinal and liver activity of MDA but a reduction in total antioxidant capacity and superoxide dismutase activity in jejunum. Vitamin E content was decreased gradually in soybean oil from fresh form until highly oxidized form. Hence, oxidation of vegetable oil with reduced antioxidant status may lead to oxidative stress and inflammation.

Effect of frying oil and polar compound derived from frying oil was investigated by Zhang et al. (16) on dextran sulfate sodium-induced colonic inflammation. It was shown that intake of frying oil could induce colitis in mice as demonstrated by increased level of inflammatory cytokines in both colon and plasma levels. Administration of polar compounds isolated from frying oil increased the severity of experimental colitis in mice. It is reasonable to postulate that the polar compounds derived from the frying oil may play a vital contributory role in the pathogenesis of inflammation as observed in this study.

## Lipid Oxidation-Derived Aldehydes

Free radicals such as reactive oxygen species (ROS) induce oxidative stress and cause cellular damage due to lipid peroxidation. There are three important steps in lipid peroxidation, namely initiation, propagation and termination. Formation of reactive aldehydes such as MDA and 4-HNE takes place when there is a catabolism of lipid hydroperoxides. These reactive aldehydes are known as second messengers of free radicals because they intensify the initial free radical chain reactions. It has been reported that MDA is more mutagenic (17) and 4-HNE is more toxic (18). Both aldehydes are involved in mediating transduction of cell signal and regulation of gene expression.

### Malondialdehyde

Under physiological condition, MDA is formed in most tissues as a by-product of prostaglandins synthesis (19). However, the levels of MDA are markedly elevated when there is a lipid peroxidation subsequent to oxidative stress. Therefore, MDA is commonly used as an indicator of lipid peroxidation. It is one of the popular biomarkers of oxidative damage and cellular injury in the field of biomedical research. MDA is a dialdehyde and readily interacts with functional groups of proteins and DNA. It is postulated that the toxicity of MDA arises from its capability to crosslink and cause mutagenesis (19). Furthermore, MDA was shown to accumulate in cells under endoplasmic reticulum (ER) stress (20), which is highly linked to cardiovascular diseases such as hypertension and atherosclerosis.

#### 4-Hydroxy-2-Nonenal

4-HNE is highly toxic due to its rapid reactions with thiols and amino groups (21). It causes significant biological complications through formation of covalent adducts in lipids, proteins and nucleic acids (21). With its high reactivity, 4-HNE has dual functions; as signaling molecule and as harmful products of lipid peroxidation. 4-HNE exerts its biological effects in the lower micromolar range of concentrations (6). Furthermore, it is the most abundant fatty acid decomposition aldehydes after oxidation of arachidonic acid (22). 4-HNE is chemotactic and may promote synthesis of cytokines (23). Studies have documented that 4-HNE may evoke immune response, eventually cause an increase in tissue injury by disturbing redox status (22, 24).

## Lipid Oxidation-Derived Aldehydes in Cardiovascular Events

### Malondialdehyde

Verma et al. (25) investigated the inflammatory marker tumor necrosis factor-alpha (TNF-α), MDA and ferric reducing antioxidant power in the blood of hypertensive patients. Findings have shown that TNF-α and MDA levels were elevated in hypertensive subjects compared to healthy controls. In contrast, serum level of ferric reducing antioxidant power was reported to be decreased. Increased oxidative stress and inflammation are implicated in the pathogenesis of hypertension.

Serinkan Cinemre et al. (26) documented that IL-4, IL-8, and IL-10 levels were significantly lower while TNF-α, and MDA levels were higher in hypertensive individuals compared to controls. Further analysis revealed that TNF-α, and IL-10 are independent predictors for grading of hypertension. Complex interaction between proinflammatory and anti-inflammatory cytokines, and oxidative stress may be involved in hypertension.

NOD-like receptor protein 3 (NLRP3) inflammasome regulates the activation of caspase-1 and induces inflammation via IL-1β (27). Activation of NLRP3 inflammasome and oxidative stress in spontaneously hypertensive rats (SHRs) was reported by Li et al. (28). Protein levels of NLRP3, caspase-1 and IL-1β in renal arteries were higher in SHRs than normotensive rats. Level of MDA was raised in SHRs. The expression of oxidative stress-related proteins, NADPH oxidase (NOX)-1, p67phox, and nuclear transcription factor-E2-related factor 2 (Nrf2) was significantly increased in SHRs. In the same study, administration of angiotensin II induced cellular injury, NLRP3 inflammasome activity and ROS formation in human umbilical vein endothelial cells (HUVECs).

There was an elevation in the serum levels of renin, angiotensin II, aldosterone, MDA and endothelin-1 levels and reduced content of nitric oxide (NO) in the SHRs (29). Furthermore, an increased expression of angiotensin II receptor type 1 in the aortic vessels with impairment of vascular relaxation in the rats were documented. Activation of renin-angiotensin-aldosterone activity causes hypertensive-induced vascular dysfunction. Wang et al. (30) reported the levels of serum lipid parameters in rats increased significantly, while high-density lipoprotein level significantly decreased in the atherosclerosis. Additionally, there was a reduced antioxidant activity and increased MDA activity. Increased expression of TNF-α, IL-1β, inducible nitic oxide synthase (NOS), and C-reactive protein, and the concentration of angiotensin II in serum, aortic, and heart tissues was observed. Expressions of inflammatory mediators and oxidative stress promote the development of atherosclerosis by increasing blood lipids in the myocardium of rats.

Vascular dysfunction mediated by homocysteine is known to be linked to oxidative stress and impaired NO system. Plasma level of MDA was elevated with a decreased NO level in rat treated with homocysteine (31). In addition, there was a deterioration of vasodilatation in rats. Mitogen-activated protein (MAP) kinases include extracellular signal-regulated kinase (ERK), c-Jun NH2-terminal kinase (JNK) and p38 MAP kinase (p38). They are important in stress-induced cellular responses including vascular remodeling. Homocysteine treatment augmented the protein phosphorylation levels of p38, ERK and JNK. Moreover, there was a downregulation of Ca^2+^, Akt, endothelial NOS and NO caused by homocysteine in HUVECs.

Oxidized low-density lipoprotein (ox-LDL) has a pro-atherogenic role in atherosclerosis. Ox-LDLs are taken-up by macrophages and transformed into lipid-laden foam cells. Ox-LDLs are cytotoxic (32) and induce apoptosis of endothelial cells and vascular smooth muscle cells (VSMCs), causing the progression of atherosclerotic plaque and development of the necrotic core (33).

Atherosclerotic lesion formation was found in apolipoprotein E-deficient mice fed with high-fat diet (34). The ox-LDL, MDA, TNF-α, and IL-1β levels were increased, whereas the superoxide dismutase level was suppressed in mice receiving high-fat diet. Concurrently, inhibitor of nuclear factor kappa B (NF-κB), IκBα protein degradation was stimulated, thus promoting the translocation of p65 subunits of NF-κB. It was reported that ox-LDL increased the levels of MDA, IL-6 and IL-1β in HUVECs (35). Toll-like receptors (TLRs) take part in inflammatory responses through activating the NF-κB signaling pathway. Zhu et al. (35) demonstrated the activation of the TLR4/NF-κB pathway in ox-LDL-treated HUVECs. Similarly, Zhao et al. (36) reported that ox-LDL induced elevation of MDA, IL-6 and TNF-α, and foam cell formation in VSMCs. Ox-LDL promoted MAP kinase pathway proteins and inhibited the expression of heme oxygenase (HO)-1 and Nrf2 protein in VSMCs. It was postulated that ox-LDL exerted its detrimental effects via ERK/Nrf2/HO-1 signaling cascade.

#### 4-Hydroxy-2-Nonenal

Renal denervation is used for reducing BP in resistant hypertension (37). However, this technique may cause endothelial injury and vascular dysfunction due to its invasiveness in nature. Su et al. (38) assessed the risk of atherosclerosis induced by renal denervation in pigs fed with high-fat diet. Expression of 4-HNE was upregulated in addition to increased activation of NF-κB. Intimal thickening with enhanced production of endothelin B receptor in VSMCs were reported. Furthermore, expression of AMPK/Akt/endothelial NOS signaling was downregulated. The obtained results suggest that treatment with renal denervation worsen endothelial dysfunction and caused intimal thickening with an increased risk of atherosclerosis in pigs receiving high-fat diet.

Increased 4-HNE protein expression in rats with metabolic syndrome, which is often associated with hypertension, hyperlipidemia, and hyperglycemia was reported (39). Metabolic syndrome induces the lipid peroxidation which leads to heart tissue changes and coronary inflammation as indicated by increased vascular cell adhesion molecule-1, IL-6 and TNF-α expression in heart. Xu et al. (40) revealed that 4-HNE contributes to the abnormal proliferation and migration of human pulmonary artery smooth muscle cells. This action may be mediated via activation of NF-κB, which led to increased right ventricular systolic pressure in monocrotaline-induced pulmonary arterial hypertension in rats.

Wakui et al. (41) documented that administration of angiotensin II increases BP and produces vascular hypertrophy. It was found that angiotensin II caused an upregulation of NOX components, including NOX4 and p22phox, and 4-HNE in aorta isolated from mice. Angiotensin II-mediated hypertension accompanied by an activation of aortic vascular p38 and JNK. The ROS-p38/JNK-dependent signaling pathway is hypothesized to be regulated in vascular pathophysiology.

Toma et al. (42) demonstrated that irreversibly glycated LDLs promote p22phox, NOX4, inducible NOS expression, and 4-HNE levels. Additionally, vascular cell adhesion molecule-1 expression and the number of monocytes adhered to human endothelial cells were found to be increased. Besides, the authors reported an increasing phosphorylation of p38 and p65 NF-κB subunits. The data suggested that oxidative stress and inflammation may mediate the endothelial dysfunction induced by glycated LDLs.

Ox-LDL-mediated NLRP3 inflammasome activation is crucial in atherosclerosis initiation and progression. Xu et al. (43) treated murine macrophages with ox-LDL. Ox-LDL induced NLRP3 inflammasome activation, with increased level of 4-HNE and ROS. Bolea et al. (44) evaluated the effects of a PUFA-rich diet on vascular integrity in apolipoprotein E mice. An increase in digestive 4-HNE production associated with a rise in plasmatic 4-HNE and ox-LDL concentrations is reported. Oxidizable n-6 PUFAs consumption is associated with a worsened endothelial dysfunction and atherosclerosis. Oxidation of n-6 fatty acids during digestion may be a key factor of vascular impairments.

Hypercholesterolemia is associated with oxidative stress and endothelial dysfunction and leads to the development of atherosclerosis. Pengnet et al. (45) revealed that a reduction of aortic NO levels, increased of aortic superoxide ion levels, led to diminished vasodilatation. Moreover, they identified an increase of lectin-type oxidized LDL receptor (LOX)-1, NADPH oxidase subunits (p47phox, NOX2, and NOX4), and inducible NOS as well as 4-HNE expression in aortic tissues from hypercholesterolemic rats. These results indicate that endothelium function in hypercholesterolemic rats was disturbed by increasing oxidative stress via upregulation of LOX-1 and NADPH oxidase.

Foam cell formation plays a vital role in the initiation and progression of atherosclerosis. Activation of aldehyde dehydrogenase 2 (ALDH2), is reported to have cardioprotective properties (46). In the presence of ox-LDL, macrophages with ALDH2 deficiency expressed reduced levels of scavenger receptor CD36. Peroxisome proliferator-activated receptor-gamma (PPAR-γ) was downregulated. 4-HNE was increased by ALDH2 deficiency and high concentration of 4-HNE suppressed the expression of PPAR-γ. These findings suggest that ALDH2 plays an important role in foam cell formation via 4-HNE/PPAR-γ/CD36 pathway (47).

**Figure 1:**
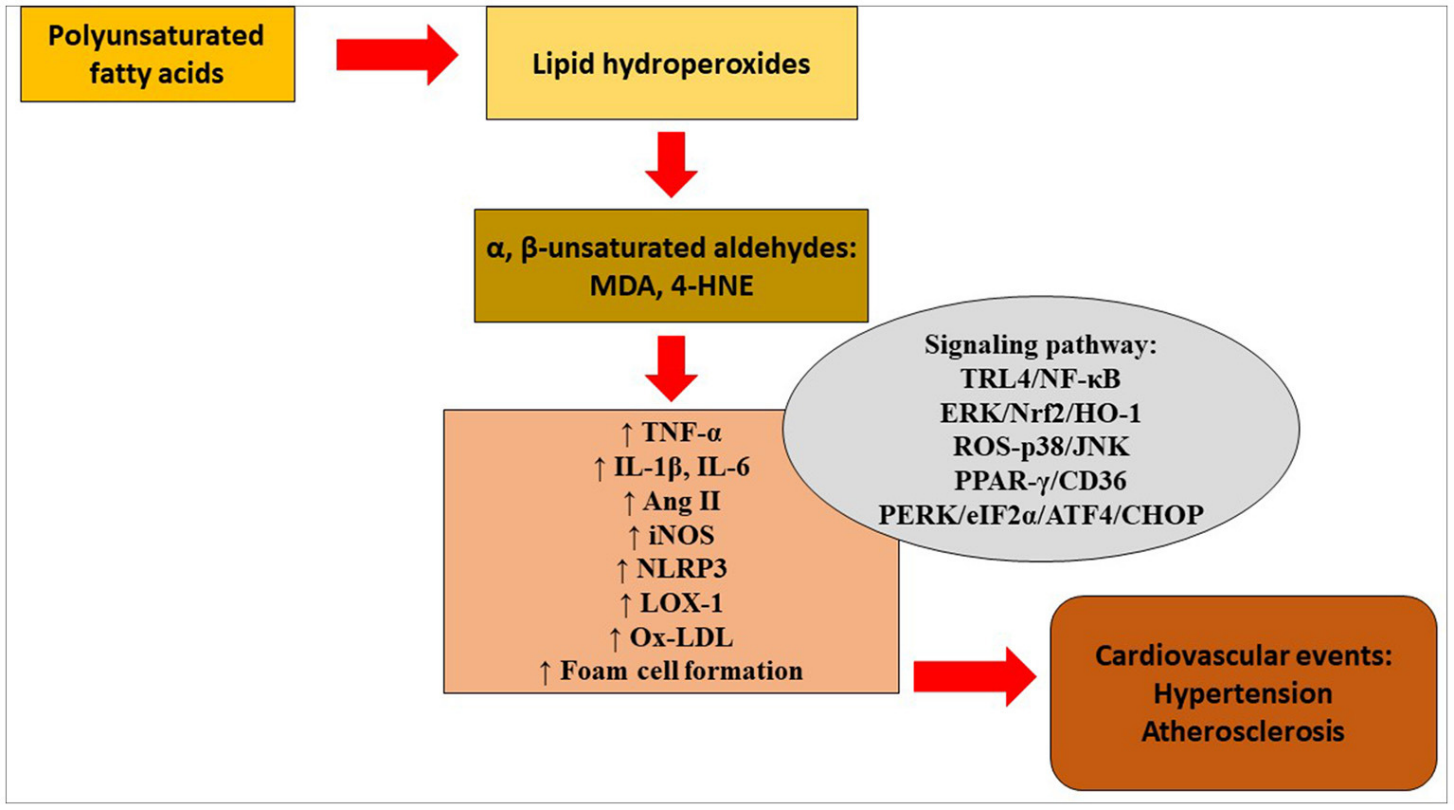
Lipid peroxidation and signaling pathways in the pathogenesis of hypertension and atherosclerosis. 4-HNE, 4-hydroxy-2-nonenal; Ang, angiotensin; ATF4, activating transcription factor 4; CHOP, CEBP homologous protein; eIF2α, eukaryotic translation initiation factor α; ERK, extracellular signal-regulated kinase; HO-1, heme oxygenase- 1; IL, interleukin; iNOS, inducible nitric oxide synthase; JNK, c-Jun NH2-terminal kinase; LOX-1, lectin-type oxidized low-density lipoprotein receptor-1; MDA, malondialdehyde; NF-κB, nuclear factor kappa B; NLRP3, NOD-like receptor protein 3; Nrf2, nuclear transcription factor-E2-related factor 2; ox-LDL, oxidized low-density lipoprotein; PERK, protein kinase R-like endoplasmic reticulum kinase; PPAR-γ, peroxisome proliferator-activated receptor-gamma; ROS, reactive oxygen species; TNF-α, tumor necrosis factor-alpha; TRL4, Toll-like receptor 4.

Serum levels of 4-HNE were significantly higher in patients with the mutant ALDH2 genotype than in patients with the wild-type ALDH2 genotype (48). Rat smooth muscle cells were treated with ox-LDL, which in turn enhanced the levels of ER markers glucose-regulated protein 78 (GRP78), protein kinase R-like ER kinase (PERK), phosphorylated eukaryotic translation initiation factor α subunit (p-eIF2α), activating transcription factor-4 (ATF-4), CEBP homologous protein (CHOP) and 4-HNE in the cells. Similarly, overexpression of ALDH2 protected smooth muscle cells against ox-LDL-induced ER stress. These findings suggest that ALDH2 may slow the progression of atherosclerosis via the attenuation of ER stress in smooth muscle cells. Figure 1 depicts lipid peroxidation and signaling pathways in the pathogenesis of hypertension and atherosclerosis.

## Anti-Inflammatory Property of Oxidized Lipids

Evidences have suggested oxidized lipids have anti-inflammatory effects (49–52). Several factors involved in determining the overall pro- or anti-inflammatory activity of oxidized lipids. The components of ox-LDL exert anti-inflammatory effects at various levels and different cellular compartments (53). Long-chain oxidized phospholipid shows anti-inflammatory and barrier protective effects on vascular endothelium, however short-chain oxidized phospholipid exhibits harmful effect (54). Early stage of ox-LDL exposure produces pro-inflammatory effect while anti-inflammatory effect is more prevalent at a later stage of exposure (55). The pro-inflammatory effect is majorly manifested through NF-κB signaling pathway (56). Low concentration of oxidized lipids activates NF-κB. In contrast, high concentration of oxidized lipid suppresses the activation of NF-κB (55). The discrepancies may be due to the multifaceted nature of oxidized lipids, as well as the source and the preparation method of oxidized lipids in different studies (33).

## Conclusion and Future Perspective

Studies involving lipid oxidation products such as MDA and 4-HNE derived from heated cooking oil on hypertension and atherosclerosis in human is limited. In addition, the exact amount of ingested lipid oxidation products would be difficult to be analyzed in human due to variability in diet and antioxidant supplementation. Furthermore, the fate of ingested lipid oxidation products in human body may be varied due to individual biological differences in metabolism. Hence, it is a challenging task for scientists to measure the consumption of lipid oxidation products. This is a research gap worth to be explored.

The use of animal model in the field of lipid oxidation research is suitable for studying the implications of lipid peroxidation *in vivo*. Besides, the animal model is an ideal platform to investigate the impact of lipid peroxidation on biomarkers in order to gain further knowledge in mechanistic role of lipid peroxidation-related diseases. Moreover, animal model is comparable to human in terms of anatomical and physiological aspects. Research animals are valuable tools for understanding the pathophysiology and in developing therapeutic interventions to control lipid peroxidation process and attenuate disease progression in humans. The major obstacle in the field of pathological processes is that it is often difficult to establish whether lipid oxidation-derived aldehydes are the cause of a disease or a consequence of the disease. More well-designed studies are required to ascertain the underlying truth.

## Author Contributions

X-FL performed literature search and wrote the manuscript.

## Conflict of Interest

The author declares that the research was conducted in the absence of any commercial or financial relationships that could be construed as a potential conflict of interest.

## Publisher's Note

All claims expressed in this article are solely those of the authors and do not necessarily represent those of their affiliated organizations, or those of the publisher, the editors and the reviewers. Any product that may be evaluated in this article, or claim that may be made by its manufacturer, is not guaranteed or endorsed by the publisher.

## Abbreviations

4-HNE: 4-hydroxy-2-nonenal
ALDH2: aldehyde dehydrogenase 2
BP: blood pressure
CFAM: cyclic fatty acid monomers
ER: endoplasmic reticulum
ERK: extracellular signal-regulated kinase
HO: heme oxygenase
HUVECs: human umbilical vein endothelial cells
IL: interleukin
JNK: c-Jun NH2-terminal kinase
LOX: lectin-type oxidized LDL receptor
MAP: mitogen-activated protein
MDA: malondialdehyde
NF-κB: nuclear factor kappa B
NLRP3: NOD-like receptor protein 3
NO: nitric oxide
NOS: nitric oxide synthase
NOX: nicotinamide adenine dinucleotide phosphate (NADPH) oxidase
Nrf2: nuclear transcription factor-E2-related factor 2
Ox-LDL: oxidized low-density lipoprotein
PPAR-γ: peroxisome proliferator-activated receptor-gamma
PUFA: polyunsaturated fatty acid
ROS: reactive oxygen species
SHRs: spontaneously hypertensive rats
TNF-α: tumor necrosis factor-alpha
TLRs: Toll-like receptors
VSMCs: vascular smooth muscle cells.
